# Variation analysis of SARS-CoV-2 complete sequences from Iran

**DOI:** 10.2217/fvl-2021-0056

**Published:** 2022-10-22

**Authors:** Jale Moradi, Parnia Moradi, Amir H Alvandi, Ramin Abiri, Mohsen Moghoofei

**Affiliations:** ^1^Department of Microbiology, Faculty of Medicine, Kermanshah University of Medical Sciences, Kermanshah, Iran; ^2^Infectious Diseases Research Center, Kermanshah University of Medical Sciences, Kermanshah, Iran

**Keywords:** Iran, lineages, mutation, phylogenetic analysis, SARS-CoV-2, S protein, variation

## Abstract

**Aim:** SARS-CoV-2 is an emerging coronavirus that was discovered in China and rapidly spread throughout the world. The authors looked at nucleotide and amino acid variations in SARS-CoV-2 genomes, as well as phylogenetic and evolutionary events in viral genomes, in Iran. **Materials & methods:** All SARS-CoV-2 sequences that were publicly released between the start of the pandemic and 15 October 2021 were included. **Results:** The majority of mutations were found in vaccine target proteins, Spike and Nucleocapsid proteins, and nonstructural proteins. The majority of the viruses that circulated in the early stages of the pandemic belonged to the B.4 lineage. **Conclusion:** We discovered the prevalence of viral populations in Iran. As a result, tracking the virus’s variation in Iran and comparing it with a variety of nearby neighborhoods may reveal a pattern for future variant introductions.

The SARS-CoV-2 virus was discovered in late December 2019 in Wuhan, Hubei province, China [1]. The disease quickly spread to other countries. According to the WHO, there were 248,467,363 confirmed cases and 5,027,183 confirmed deaths up until November 2021 [2].

*ORF1ab*, *ORF2*, *ORF3abcd*, *ORF3b*, *ORF3c*, *ORF3d*, *ORF4*, *ORF5*, *ORF6*, *ORF7a*, *ORF7b*, *ORF8*, *ORF9a*, *ORF9b*, *ORF9c* and *ORF10* comprise the SARS-CoV-2 genome. These open reading frames (ORFs) encode four structural proteins – Spike (S) glycoprotein, Envelope (E), Membrane (M) and Nucleocapsid (N) proteins – as well as nine accessory proteins and 16 nonstructural proteins (NSP1–16) [3]. Viruses can change and spread quickly around the world due to various selective pressures [4]. As a result, numerous studies are being conducted to identify virus variations in various geographical regions [4,5]. Changes in these parts of the genome may affect virulence and transmissibility, because some viral proteins are targets for neutralizing antibodies [6]. Furthermore, mutations can impair vaccine efficacy and the validity of diagnostic tests due to changes in the targeted sequences [7,8].

In the middle of October, there are currently 4,924,450 full genome sequences of SARS-CoV-2 in Global Initiative for Sharing All Influenza Data (GISAID), and the number of sequences submitted is increasing. GISAID [9,10], Nextstrain [10] and Phylogenetic Assignment of Named Global Outbreak Lineages (Pangolin) [11] are SARS-CoV-2 nomenclature systems. The GISAID nomenclature system identified significant clades based on marker mutations within eight high-level phylogenetic categories. Early in the pandemic two clades, S and L, were introduced, followed by the evolution of L into V and G; and then G into GH, GR and GV; and more recently, GR into GRY [12]. The Pangolin nomenclature system is more inclusive of phylogenetic clades and has the potential to supplement two other nomenclature systems (NextStrain and GISAID) [13].

Previous molecular evolutionary studies on the SARS-CoV-2 genome revealed that purifying selection and recombination played a role in the virus’s origin from nonhuman mammalian reservoirs (bats) [14]. Mutations, as well as their monitoring, are critical because they cause changes in viral pathogenicity [15].

We focused on mutations in vaccine target proteins such as S, N, M and E in this study. In addition, we examined the entire SARS-CoV-2 genome in 280 Iranian samples submitted to GISAID to determine genetic distance and mutation rate.

## Materials & methods

### Data retrieval & preprocessing

The data were retrieved from GISAID. By 15 October 2021, 280 SARS-CoV-2 complete sequences with high coverage related to Iran were released in GISAID. GISAID has defined ‘complete sequences’ as genomes >29,000 bp in length, with high coverage considered to be sequences with 1% undetermined characters, 0.05% unique amino acid mutations and no insertion/deletions verified in advance by the submitter. We downloaded all of the sequences and filtered for high-quality sequences, removing two sequences due to the large number of gaps. The remaining 278 sequences were then trimmed because the sequencing did not begin and end at the same point for all sequences. We analyzed 278 SARS-CoV-2 genomes with 29,647 nucleotide length, with nearly half of the samples collected in Tehran (Iran’s capital), 18% from northern cities, 9% from the south, 8% from the center of Iran, 6 and 4% from eastern and western cities, respectively, and around 5% from unknown sources (Supplementary Table 1).

### Alignment, nucleotide & amino acid variations

All 278 sequences were aligned to the SARS-CoV-2 reference genome (NC 045512.2) using MAFFT (v.7.455) [16]. From a multiple sequence alignment file, single-nucleotide polymorphism sites were used to extract genome variations [17]. The relationship of each variation to SARS-CoV-2 genome ORFs was then investigated [3]. Nextstrain was also used to analyze the genomes and compare the results [10].

### Phylogenetic analysis

The multiple sequence alignment file was visualized for phylogenetic analysis using the UGENE software for quality control and trimming [18]. The trimmed file was used to build the phylogenetic tree using the maximum likelihood method and RaxML-NG v.0.9.0 (100 bootstraps) [19]. The constructed tree was visualized using iTOL [20]. Pangolin [11] was used to classify the sequences.

## Results

### Nucleotide variations in SARS-CoV-2 sequences

**Figure 1.**
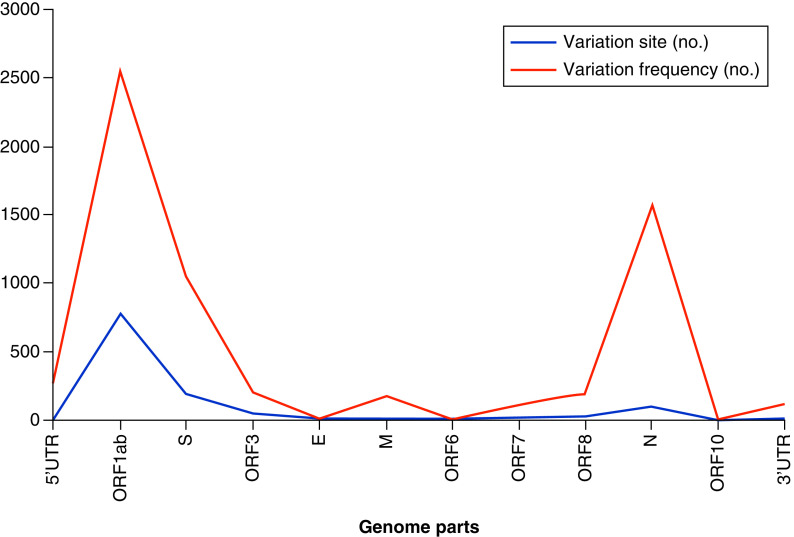
The number of nucleotide variations in SARS-CoV-2 isolates from Iran between January 2020 and October 2021. The blue line represents the number of variation types, while the red line represents the total number of variations. E: Envelope; M: Membrane; N: Nucleocapsid; ORF: Open reading frame; S: Spike; UTR: Untranslated region.

In comparison to the reference genome, 6231 variations were observed in 1289 variation sites in SARS-CoV-2 genomes (Supplementary Table 2). The majority of the variations were found in the *ORF1ab*, *S* and *N* regions, which had 787, 194 and 102 variation sites, respectively (Figure 1).

### Phylogenetic analysis

**Figure 2.**
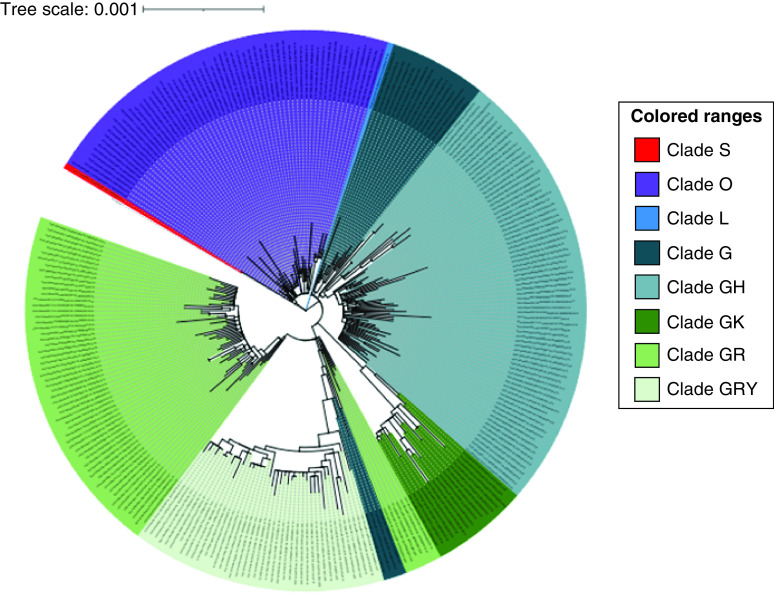
Phylogenetic analysis of 278 SARS-CoV-2 whole genomes from Iran. Maximum likelihood phylogenetic tree was built using RaxML-NG and visualized using *iTOL*. Pangolin was used to perform lineage assignment.

**Figure 3.**
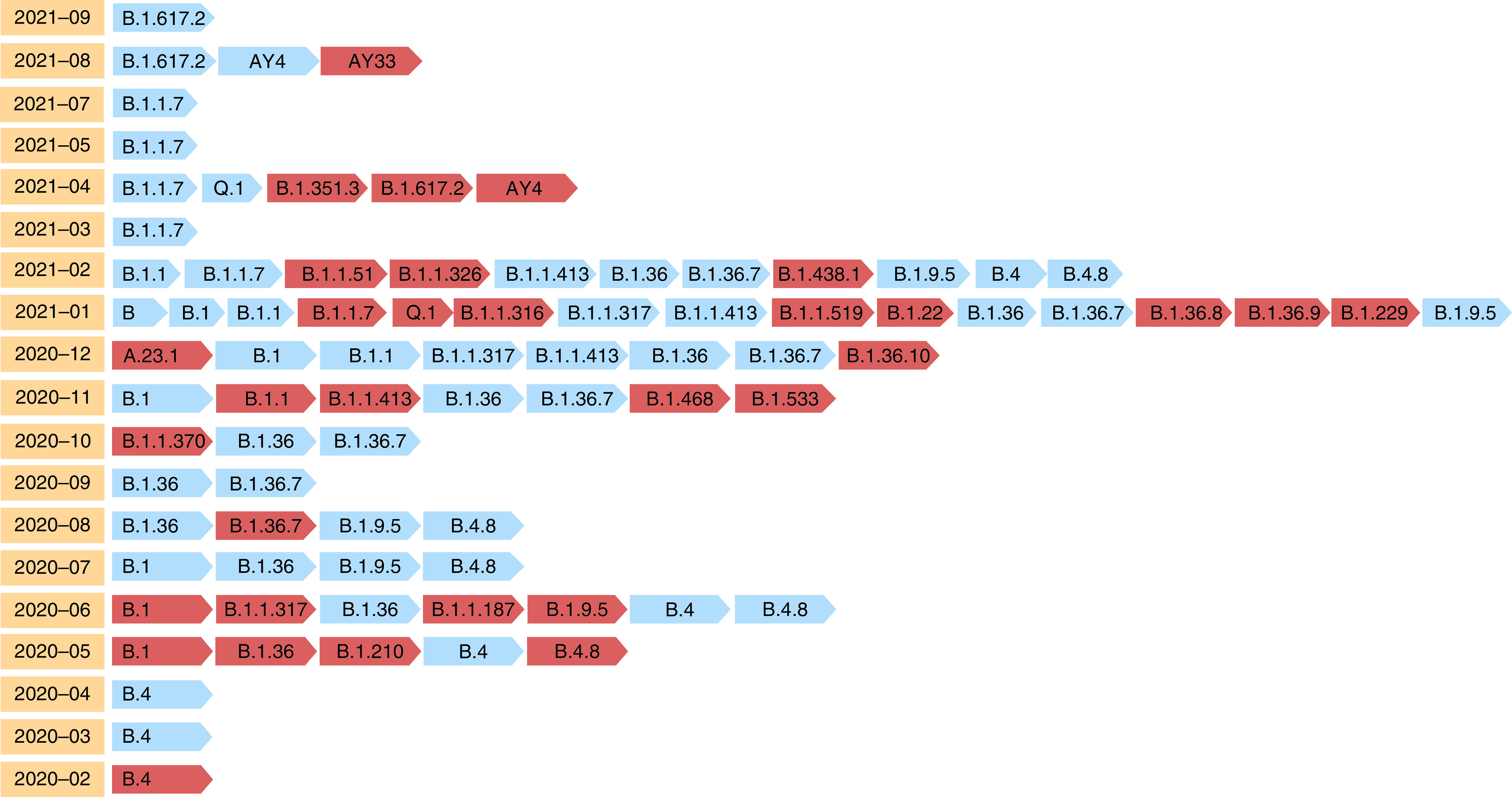
Distribution of SARS-CoV-2 variants in Iran between January 2020 and October 2021. Variants are arranged chronologically on the Pangolin website, with new variant introductions denoted by a red arrow in each timeline.

The majority of the genomes in the current study belonged to the clades GH, GR, GRY and O (which is a general clade containing sequences not classified by GISAID) (Figure 2). According to Pangolin analysis, new lineages have emerged during the epidemic. The new lineages caused the frequencies of the other lineages to shift, and they were classified as frequent lineages until the next and new lineages emerged (Figure 3). In the early stages of the pandemic, B.4 was the dominant lineage in Iran from the beginning of 2020 to the middle of the same year. However, the frequency of the B.4 lineage was gradually reduced by the emergence of new lineages, which were then replaced by the other frequent lineages. After B.4, the B.1.36 and B.1.36.7 lineages were the most common from the middle of 2020 to the beginning of 2021. The B.1.617.2 lineage was detected in April 2021 and has been spreading in recent months, while the B.1.1.7 lineage was the most common in 2021. Furthermore, our findings suggested that AY.4 could have evolved alongside B.1.617.2 or independently as the second most common lineage.

### Mutations in SARS-CoV-2 proteins

**Figure 4.**
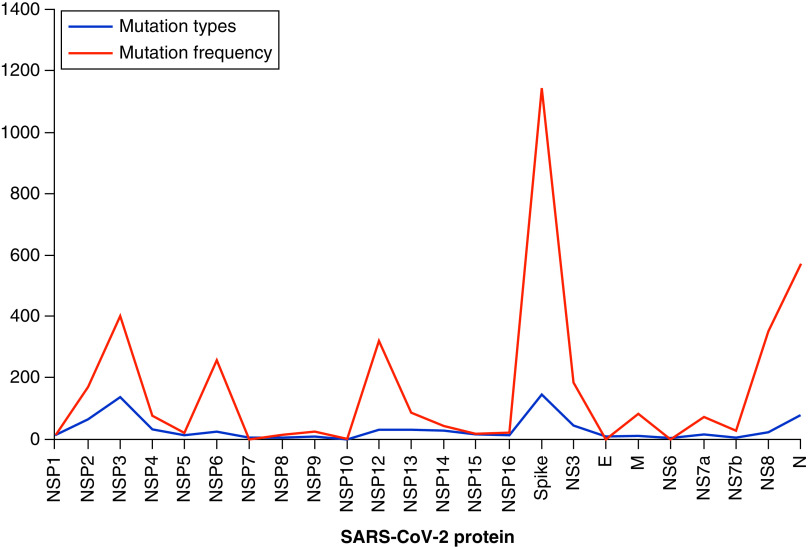
The mutation profile of 278 SARS-CoV-2 genomes from Iran between January 2020 and October 2021. The blue line represents the number of mutation types, while the red line represents the total number of mutations. The most mutations were found in the Spike and Nucleocapsid proteins. E: Envelope; M: Membrane; N: Nucleocapsid.

In total, 3955 amino acid changes (786 mutation types) were detected in the protein sequences of all 278 SARS-CoV-2 genomes (Supplementary Tables 3 & 4). Nonstructural proteins (NSP1–16), S protein, E protein, M protein, N protein and four accessory proteins (NS3, NS6, NS7 and NS8) all had mutations. The most mutations were found in NSP1–16 and S proteins. In particular, 1144, 578, 83 and seven mutation types were found in vaccine target proteins S, N, M and E, respectively (Table 1). The most common S protein mutations were D614G, I210del, S477N, D138Y, Y144del, A570D, T716I and H69del. The most common mutations in the N protein were R203K, G204R, S194L, D3L and S235F; I73M and I82T mutations in the M protein were also common. The relationship between the number and types of mutations was investigated (Figure 4). In NS6, E, NS7b and some nonstructural proteins in *ORF1ab*, the number of mutation events and mutation type values were nearly equal, but they were far apart in other proteins.

**Table 1.** The number and types of mutations in vaccine target proteins.

S	No.	S	No.	N	No.	M	No.	E	No.
I210del	64	A706V	1	R203K	110	I73M	56	V14del	1
D614G	224	T20A	1	G204R	111	I82T	16	T9I	2
S477N	57	Q675R	1	S194L	67	T208S	2	V62F	1
D138Y	56	D936Y	2	D3L	46	T175M	1	P71L	1
L5F	4	D1163Y	1	S235F	55	G78D	1	S68F	1
H69del	41	T998P	1	D63G	15	L29F	1	L73F	1
T716I	42	E180K	2	R203M	16	H155Y	1		
A570D	43	M1237I	1	G215C	12	H125N	1		
Y144del	50	Q52H	1	P326L	1	T208I	1		
G142D	14	D253G	1	A220V	7	A2S	1		
D1118H	42	V1264L	1	P13T	1	L34F	1		
N501Y	42	Y837H	1	A35V	2	S197T	1		
D950N	17	Q613H	1	G34W	8				
L699I	17	R78M	1	P162S	1				
P681H	42	Q677H	2	S188P	1				
V70del	41	D1260H	1	D377Y	15				
S982A	43	I68T	1	P326S	1				
I100T	17	K1191N	2	T205I	1				
T95I	12	K1205R	1	L219F	1				
P681R	17	D80Y	1	D401Y	2				
T478K	16	D215Y	1	M234I	15				
T19R	16	C1248F	1	D3Y	7				
F157del	15	T859N	1	A208del	1				
L452R	14	A771S	2	R209del	1				
R158del	15	A1020S	1	A211del	1				
E156G	15	G261K	1	M210del	1				
T572I	4	H146Q	1	P207del	1				
E1202Q	5	Q14H	1	A119S	1				
W104C	6	A27V	1	G178V	1				
L244del	2	G1219C	1	E378Q	1				
F1256L	2	R214P	1	S193I	1				
H655Y	3	Y144H	1	A35E	1				
A263V	1	Q675R	1	S186F	7				
Q675H	5	N709K	1	K248N	1				
D215G	2	V143D	1	K248N	1				
Q23K	1	L18F	1	A252S	2				
N1023B	1	A163T	1	N192K	5				
P479L	1	R78K	1	Q39E	1				
D574Y	2	G181A	1	A211V	1				
K77T	1	A163T	1	P13T	1				
L1141W	1	F490S	2	A308S	1				
K417N	1	Y365C	1	R209I	1				
E484K	1	P1162S	1	N192K	1				
L242del	2	T859N	1	P13L	1				
A701V	1	Q675R	1	A90S	1				
D80A	2	I1130M	1	R209G	1				
A243del	2	D1260Y	1	E290Q	1				
H69Y	1	P1069T	1	N196I	2				
V1122L	1	D80Y	1	P80L	2				
T859I	1	D1145E	1	A273S	2				
M177I	2	V483A	1	S202N	1				
H655R	1	S939F	1	K375N	2				
E1150D	1	I850L	2	A173V	2				
D1199N	1	T250I	1	G243C	1				
A222V	5	T299I	1	G212C	1				
V976F	1	T29A	1	T417I	1				
C1250R	1	A262S	1	A414S	2				
D1257E	1	N1192S	1	A211V	2				
S255F	1	D808G	1	R195I	1				
L938F	1	H146R	1	G25C	2				
K417N	1	A344S	1	Q239L	1				
E484K	1	M1229I	1	Q389H	2				
A701V	1	S929I	1	T16M	2				
V826M	1	Q314R	1	D216E	1				
Q675H	5	D574N	1	R209I	1				
I472V	1	G142S	1	A211T	1				
H49Y	1	A846V	1	L221F	1				
A1070S	1	A845S	1	A35V	1				
G181V	1			P13T	1				
T678S	2			D144Y	2				
L54F	1			G19R	1				
D138H	1			D103Y	1				
K558N	1			P13T	6				
S151I	1			T24I	1				
T732I	1			T296I	1				
S704L	1			T326I	1				
E1150A	1			P383L	1				
T22I	7			S201I	1				

E: Envelope; M: Membrane; N: Nucleocapsid; S: Spike.

## Discussion

Iran reported the first confirmed cases of COVID-19 infection in Qom on 19 February 2020 [21]. In Iran, there have been five major COVID-19 waves [22]. Previous studies demonstrated that there were different epidemiological characteristics in the different waves of the COVID-19 outbreak; for example, the epidemiological characteristics observed in the first wave of the outbreak were distinct from those observed in subsequent waves [23–25]. The mortality rates from the first to fifth peaks were 14.4, 18.2, 23, 9.02 and 9.4%, respectively [26]. The third peak had the most respiratory involvement, and the fourth peak had the least respiratory involvement, but the need for special care or tracheal intubation was greater in the first wave of the disease [26]. Cough and abdominal pain were more prevalent in the first wave, decreased in the second, then resurfaced in the third [27]. The second wave of the disease also included gastrointestinal symptoms [24,26,27].

Analysis of SARS-CoV-2 whole genome sequencing data from five waves of the pandemic revealed differences in the majority of circulating clades in Iran. V and L clades were discovered during the first wave; G, GH and GR clades identified the second wave; GH and GR were circulating clades during the third wave; and GRY (α variant), GK (δ variant) and one GH clade (β variant) were discovered in the fourth wave. All of the viruses in the fifth wave belonged to the clade GK (δ variant) [28]. The genomes in the current study were mostly from the clades GH (25%), GR (23%), O (21%) and GRY (14%). A recent study on SARS-CoV-2 whole genome analysis in the Asian continent (including ten countries: India, Bangladesh, Pakistan, Sri Lanka, China, Japan, Malaysia, Iran, Thailand and Saudi Arabia) found that the frequency of the clades was GH > GR > O > G > S > L > V, which is consistent with our findings [29]. Clade O was introduced as the most frequent in the mentioned study in Iran, Sri Lanka, Thailand, China and Malaysia, but our analysis revealed that GH is a more frequent clade than O at the time of study, possibly because the date of our study was later than that of the previous study.

SARS-CoV-2’s genome contains seven major ORFs and 23 unannotated ORFs [3,30]. *ORF1ab* is composed of two overlapping ORFs (*ORF1a* and *ORF1b*), which take up two-thirds of the viral genome and encode a polypeptide that is cleaved into 16 nonstructural proteins. According to our findings and many other reports, the majority of the variation sites are raised in *ORF1ab* [5]. Other important ORFs code for four canonical structural proteins: S, M, E and N. Because vaccine target protein amino acid sequences must be conserved, it is critical to characterize the type and rate of mutations in these proteins [31,32]. Our findings show that S and N proteins accounted for approximately 29 and 15%, respectively, of the total number of mutation events. Furthermore, 38% of the mutations were found in NSP1–16. Other studies that support our findings show that S and N proteins have a high mutation rate, following *ORF1ab* [5]. However, in our experience, when comparing the same studies, mutations in intergenic sites are much more common.

From the beginning of 2020 to the middle of the year, B.4 was the most common lineage in Iran, indicating that the pandemic was still in its early stages. However, the emergence of new lineages gradually reduced the frequency of the B.4 lineage, which was then replaced by the other frequent lineages. From the middle of 2020 to the beginning of 2021, the B.1.36 and B.1.36.7 lineages were the most common after B.4. Despite the recent spread of the B.1.617.2 lineage, the B.1.1.7 lineage was the most common in 2021. The B.1.1.7 lineage was discovered for the first time in the UK in 2020 [33]. B.1.617.2 is another significant lineage that was discovered in India in late 2020 [34]. In comparison to D614G (wild-type Wuhan), the B.1.617.2 lineage exhibited two significant differences: increased resistance to neutralizing antibodies from recovered patients and decreased sensitivity to vaccine-induced antibodies [35]. Furthermore, our findings suggested that the AY.4 (B.1.617.2 lineage) could be expanded alongside or independently as the second most common lineage.

The most important mutations (in the S protein) in this variant are: N501Y replacement leading to increased ACE2 binding; P618H replacement leading to a furin cleavage site; and Δ69–70:, leading to the failure of molecular tests to diagnose the virus [36–38]. Most mutations were found in NSP1–16 and S proteins, according to our findings. In particular, 1144, 578, 83 and seven mutation types were found in vaccine target proteins S, N, M and E, respectively. The most common mutations in the S protein were D614G, I210del, S477N, D138Y, Y144del, A570D, T716I and H69del. A total of 80 variants and 26 glycosylation mutants of the S protein were tested for infectivity and reactivity in a systematic review [39]. Most of the variants were susceptible to neutralizing antibodies, but combinations of D614G with some variants such as A475V, L452R, V483A and F490L were unidentifiable by neutralizing antibodies and more infectious [39–41]. The S protein D614G mutation was found in 26% of the sequences studied in March 2020, but it had increased to 74% by June 2020 [42].

The S477N mutant is resistant to neutralization antibodies (receptor binding domain-targeting), but when convalescent serum was used, it behaved similarly to the wild-type [43]. Furthermore, the S477N mutation increases ACE2 receptor affinity [37]. Some studies have linked A570D, T716I and other mutations (P681H, S982A, D1118H, N501Y in lineage B.1.1.7) to a higher viral load [44]. Some significant deletion mutations (e.g., V70del, Y144del and H69del) may play a role in SARS-CoV-2 pathogenesis by allowing the virus to interact with lung receptors more effectively or by becoming a target of neutralizing antibodies [45].

The most common mutations in the N protein found in this study were R203K, G204R, S194L, D3L and S235F. Mutations I73M and I82T in the M protein were also common. The R203K mutation may not affect N protein function because the mutant protein contains the same positively charged arginine and lysine residues [46]. According to global sequence analysis, R203K was found in 37.3% of the sequences and G204R was found in 37% of the sequences. S194L was also reported in other countries, including England and Scotland (3.2 and 39%, respectively), the USA (29%) and India (11%) [47]. Previous research suggested that the I82T mutation could confer a biologically selective advantage. In contrast, the combination of this mutation with P681H, T716I and S494P (S mutations) may cause a specific concern [48]. Overall, viral protein modifications (M and E) may be important in altering signaling pathways and increasing SARS-CoV-2 pathogenesis via the hijacking of host cellular machinery and rapid internalization [49].

## Conclusion

In summary, the current study collected Iranian SARS-CoV-2 genomes, analyzed them with bioinformatics and validated the results with online platforms, reviewing SARS-CoV-2 sequences discovered in Iran. The majority of the variations were related to *ORF1ab*, which is involved in virus replication and transcription, according to our findings. Lineage B.4 was discovered to be the most common variant in Iran. However, the frequency of the B.4 lineage gradually decreased and was then replaced by the other frequent lineages. Although the B.1.617.2 lineage has been spreading in recent months, the B.1.1.7 lineage was the most common in 2021. These findings, along with similar future research, provide an opportunity to track and predict transmission behavior patterns in order to implement appropriate pandemic control strategies in Iran. Furthermore, we discovered some significant mutations that have the potential to alter the disease’s epidemiological patterns. As a result, tracking the virus’s variation in Iran and comparing it with a variety of nearby neighborhoods may reveal a pattern for future variant introductions. Iran’s neighbors, as well as the rest of the world, will be affected by these surveillance policies.

## Future perspective

These data, along with similar future studies, will enable researchers to track and predict transmission behavior patterns in order to apply appropriate pandemic control strategies in Iran in the future.

**Summary points**

**Background**

- SARS-CoV-2 is a new emerging single-stranded RNA virus that was first reported in late December 2019 in Wuhan, Hubei province, China.
- The disease spread quickly to other countries.
- SARS-CoV-2 genome sequences have been deposited in the ‘Global Initiative for Sharing All Influenza Data’ public database, and phylogenetic analysis can be used to characterize the virus’s geographical evolution pattern.

**Methods**

- A total of 278 complete SARS-CoV-2 sequences associated with Iran had been made public up to 15 October 2021.
- Single-nucleotide polymorphism sites were used to extract genome variations from a multiple sequence alignment file.
- The relationship between each variation and SARS-CoV-2 genome open reading frames was then investigated.
- A phylogenetic tree was constructed using the trimmed multiple sequence alignment file.

**Results**

- In total, 3955 amino acid changes (786 mutation types) were found in the protein sequences of all 278 SARS-CoV-2 genomes that were retrieved.
- According to our findings, the majority of mutations were found in nonstructural (NSP1–16) and Spike proteins.
- In the early stages of the pandemic, B.4 was the dominant lineage in Iran, but was then supplanted by other common lineages such as B.1.36, B.1.36.7, B.1.1.7, B.1.617.2 and AY.4.

**Discussion & conclusion**

- The study investigated genomic variations, protein mutations and the phylogeny of SARS-CoV-2 sequences in Iran.
- Significant lineages were discovered with mutations in the Spike, Nucleocapsid, Membrane and Envelope proteins.
